# AWARE A novel web application to rapidly assess cardiovascular risk in type 2 diabetes mellitus

**DOI:** 10.1007/s00592-023-02115-x

**Published:** 2023-06-04

**Authors:** Cesare Berra, Roberto Manfrini, Marco Mirani, Loredana Bucciarelli, Ahmed S. Zakaria, Sara Piccini, Renata Ghelardi, Maria Elena Lunati, Sylka Rodovalho, Francesco Bifari, Paolo Fiorina, Franco Folli

**Affiliations:** 1 Department of Endocrinology, Nutrition and Metabolic Diseases, IRCCS Multimedica, Milan, Italy; 2 Departmental Unit of Diabetes and Metabolism, San Paolo Hospital, ASST Santi Paolo E Carlo, Milan, Italy; 3 Endocrinology and Metabolism, Department of Health Science, Università Degli Studi Di Milano, Milan, Italy; 4 IRCCS Humanitas Research Hospital, Rozzano, Milan Italy; 5 UOC Coordinamento E Integrazione Rete ASST Melegnano E Della Martesana, Melegnano, Milan, Italy; 6 Division of Endocrinology, ASST Fatebenefratelli-Sacco, Milan, Italy; 7 Endocrinology and Metabolism, Pontificia Università de Campinas, Campinas, Sao Paulo State, Brazil; 8 Laboratory of Cell Metabolism and Regenerative Medicine, Department of Medical Biotechnology and Translational Medicine, University of Milan, LITA, Segrate, Italy; 9 International Center for T1D, Pediatric Clinical Research Center Romeo Ed Enrica Invernizzi, DIBIC, Università Di Milano, Milan, Italy; 10 Nephrology Division, Boston Children’s Hospital, Harvard Medical School, Boston, MA USA

**Keywords:** Type 2 diabetes mellitus, Cardiovascular risk, Digital tools, Glucagon like peptide 1 receptor agonists, Sodium-glucose co-transporter-2 inhibitors

## Abstract

**Aim:**

To describe the development of the AWARE App, a novel web application for the rapid assessment of cardiovascular risk in Type 2 Diabetes Mellitus (T2DM) patients. We also tested the feasibility of using this App in clinical practice.

**Methods:**

Based on 2019 European Society of Cardiology/European Association for the Study of Diabetes criteria for cardiovascular risk stratification in T2DM, the AWARE App classifies patients into very high (VH_CVR_), high (H_CVR_) and moderate (M_CVR_) cardiovascular risk categories. In this retrospective clinical study, we employed the App to assess the cardiovascular risk of T2DM patients, while also collecting data about current glycaemic control and pharmacological treatment.

**Results:**

2243 T2DM consecutive patients were evaluated. 72.2% of the patients were VH_CVR_, 8.9% were H_CVR_, 0.8% were M_CVR_ while 18.2% did not fit into any of the risk categories and were classified as “moderate-to-high” (MH_CVR_). Compared with the other groups, patients with VH_CVD_ were more frequently ≥ 65 years old (68.9%), with a longer disease duration (≥ 10 years [56.8%]), a history of cardiovascular disease (41.4%), organ damage (35.5%) and a higher numbers of cardiovascular risk factors. Patients with MH_CVD_ generally had disease duration < 10 years (96%), younger age (50–60 years [55%]), no history of cardiovascular disease, no organ damage, and 1–2 cardiovascular risk factors (89%). Novel drugs such as Glucagon Like Peptyde 1 Receptor Agonists or Sodium-Glucose Linked Transporter 2 inhibitors were prescribed only to 26.3% of the patients with VH_CVR_ and to 24.7% of those with H_CVR_. Glycaemic control was unsatisfactory in this patients population (HbA1c 7.5 ± 3.4% [58.7 ± 13.4 mmol/mol]).

**Conclusions:**

The AWARE App proved to be a practical tool for cardiovascular risk stratification of T2DM patients in real-world clinical practice.

**Supplementary Information:**

The online version contains supplementary material available at 10.1007/s00592-023-02115-x.

## Introduction

Type 2 Diabetes Mellitus (T2DM) is a global health emergency. Its incidence and prevalence are exponentially increasing, particularly in developing countries [1].

T2DM micro- and macrovascular complications contribute substantially to the burden of the disease. They are major causes of increased morbidity and mortality, frequently resulting also in clinical and surgical emergencies [2, 3]. Clinical and epidemiological data have demonstrated that T2DM increases by 3–4 folds atherothrombotic risk, as well as chronic kidney disease risk [4, 5]. In T2DM, cardio-renal events are further boosted by the pathogenic link between ischemic heart disease and heart failure with renal disease [6].

Preventive and pharmacological interventions are essential to limit the growing burden of T2DM-related cardiovascular (CV) complications [7, 8]. In the last decade two new classes of anti-diabetic drugs proved to be particularly effective in attaining these goals: the Glucagon Like Peptide 1 Receptor Agonists (GLP-1 RA) and the Sodium-Glucose Co-Transporter-2 inhibitors (SGLT2i) [9–21]. The benefits of these two classes of drugs are not limited to glycaemic control since they also proved to be effective in reducing blood pressure [21, 22], body weight [23] and sub-inflammation [24, 25].

Clinical trials results with GLP-1 RA and SGLT2i have changed T2DM treatment paradigms, shifting the therapeutic target from glycaemic control to the possible prevention/slowing of organ damage and increased survival. This change of perspective has been implemented in recent guidelines developed jointly by diabetologists and cardiologists. In the European Society of Cardiology (ESC)/European Association for the Study of Diabetes (EASD) and in the ESC/European Association of Preventive Cardiology (EAPC) guidelines, GLP-1 RA and SGLT2i are included in pharmacological management algorithms as first-line treatment for patients with high or very high CV risk [26, 27].

This new approach requires a prompt and accurate assessment of each T2DM patient’s CV risk, which should then guide physicians in the choice of the best treatment options. Moreover, since CV risk can be regarded as a continuum, constantly changing over time, this risk should be assessed not only at the onset of the disease but also regularly during patients’ lifetime [28, 29]. This will allow taking into account the occurrence of intercurrent events (such as chronic diseases, cognitive decline, aging, coronary artery disease, stroke and loss of kidney function) which could change the CV risk level and thus prompt a treatment modification.

To guide physicians in assessing patients’ CV risk, the 2019 ESC-EASD guidelines introduced CV risk stratification criteria [26]. These guidelines proposed three risk categories (very high, high, and moderate) based on the presence of CV disease, other target organs damage (proteinuria, renal impairment defined as eGFR < 30 mL/min/1.73 m^2^, left ventricular hypertrophy, retinopathy), duration of disease, age, and/or presence of other known risk factors (arterial hypertension, dyslipidemia, smoking, obesity) [26].

Although ESC-EASD criteria are straightforward, their implementation may result unpractical in everyday clinical routine. This is particularly true in current diabetological practice landscape, where large numbers of patients and tight visit times may result in underutilization of this CV stratification tool and delay of organ-protective therapies initiation [30].

Technology may help overcome this obstacle. Today it is increasingly easy and unexpensive to build tailored small pieces of software which can simplify the execution of time-consuming tasks.

To this end, we developed the new AWARE App, a Web application that allows to assess CV risk according to the ESC/EASD criteria in about 20 s. The App was employed by a network of hospitals and outpatient clinics in Lombardy (Italy) to raise awareness about CV risk in T2DM and to simplify patient categorization.

This study aimed to test the feasibility of AWARE App use in routine clinical practice while also collecting real-world data about CV risk, glycaemic control and pharmacological treatment of patients with T2DM.

### Methods

#### The AWARE app

AWARE is a Web App which runs on a Web server and can be loaded by any Internet browser, using both personal computers and mobile devices (smartphones, tablets). The AWARE App was developed by the Italian software house SoftwareVM on behalf of the Diabetes Centers involved in this study.

We named the App “AWARE” (raise AWAREness on the importance of CV risk assessment in T2DM patients) since we hypothesized that its use could help to choose the most appropriate antidiabetic medication according to each patient cardiovascular risk.

The main function of the AWARE App is the assessment of CV risk based on 2019 ESC/EASD criteria (Table 1). After loading, the App shows the main screen, which includes several options concerning the AWARE Project (Risk assessment, Patient report, Documents, About the project, and Working group). Currently, the only active option is “Risk assessment”: by clicking/touching it the CV risk assessment section is loaded. It consists in a short form which must be filled with some patient’s information such as age (< 50 or ≥ 50 years old), diabetes duration (< 10 or ≥ 10 years), presence of established CV disease (yes or no), organ damage (proteinuria, kidney disease, retinopathy, or LVH) and CV risk factors (smoke, dyslipidemia, hypertension, obesity, age; Supplementary Fig. 1). Once the form is filled, a button named “Assess risk” activates: by clicking/touching it, the App calculates and displays the patient’s level of CV risk. Only the age and the diabetes duration are mandatory informations required by the App; the other fields should be filled based on patient’s characteristics, but do not prevent the activation of the “Assess risk” button. The completion of the AWARE App Risk assessment form requires about 20 s, making the CV risk assessment fast and easy.

**Table 1:** Cardiovascular risk categories in patients with diabetes according to the 2019 ESC/EASD recommendations

Very high risk	Patients with DM and established CVD or other target organ damage
	or three or more major risk factors
	or early onset T1DM of long duration (> 20 years)
High risk	Patients with DM duration ≥ 10 years without target organ damage plus any other additional risk factor
Moderate risk	Young patients (T1DM aged < 35 years or T2DM aged < 50 years) with DM duration < 10 years, without other risk factors

CV: cardiovascular; CVD: cardiovascular disease; DM: diabetes mellitus; T1DM: type 1 diabetes mellitus; T2DM: type 2 diabetes mellitus. Other target organ: proteinuria, renal impairment defined as eGFR < 30 mL/min/1,73 m2, left ventricular hypertrophy, or retinopathy. Major risk factors: age, hypertension, dyslipidemia, smoking, or obesity. Adapted from Cosentino F, et al. Eur Heart J. 2020;41(2):255–323 [26]

The AWARE App used in this study also recorded the patients’ level of glycated haemoglobin (HbA1c) and the prescribed class of medication, to provide further opportunities for data interpretation and future studies.

The AWARE App is free and available online in English language at the following URL: https://aware.softwarevm.online/** (user ID: Aware; password: Aware).**

#### Study design and participants

This was a retrospective, observational, multi-center study, conducted by a network of Diabetes Centers in Lombardy (Italy). 2243 consecutive T2DM patients attending the Centers from November 2020 to April 2021 were enrolled. The AWARE App was used to calculate each patient’s CV risk and to record his/her HbA1c level and current pharmacological treatment. Anonymized data were stored on the App Web server and retrospectively analysed. The study protocol was approved by the IRCSS MultiMedica, Sesto San Giovanni (MI), Italy, Ethics Committee (Protocol n. 498.2021 approved on 10/03/2022).

#### Statistical analysis

Continuous variables are reported as mean and standard deviation (SD), while categorical variables are reported both as absolute numbers and percentages representing relative prevalence. Differences between groups were analysed with the Chi-squared test or Fischer's exact test for categorical variables and with Student t-test or Mann–Whitney U-test for continuous variables, as appropriate. A one-way analysis of variance (ANOVA) was performed to compare differences among groups in continuous variables. A *P* value < 0.05 was considered statistically significant. All the analyses were performed with STATA 12.1 (Statistics/Data Analysis, Stata Corp, College Station, Texas).

## Results

Overall, 2243 T2DM patients underwent CV risk assessment with the AWARE App and were included in this analysis. The majority of these subjects (n = 1619 [72.2%]) had a very high CV risk (VH_CVR_), 199 (8.9%) a high CV risk (H_CVR_), and only 17 (0.8%) a moderate CV risk (M_CVR_) (Table 2).

**Table 2:** Clinical characteristics of the patients included in the study (N = 2,243), by risk categories

	CV risk category				
	Moderate	Moderate-to-High	High	Very high	*P* VALUE
Patients per risk category	17 (0.8%^a^)	408 (18.2%^a^)	199 (8.9%^a^)	1619 (72.2%^a^)	< 0.001
History of established CVD	0 (0%)	0 (0%)	0 (0%)	671 (41.4%)	< 0.001
Diabetes duration ≥ 10 years	0 (0%)	18 (4.4%)	0 (0%)	919 (56.8%)	< 0.001
Age					< 0.001
< 50 years	17 (100%)	112 (27.5%)	10 (5.0%)	108 (6.7%)	
≥ 50 years	0 (0%)	296 (72.5%)	189 (95%)	1511 (93.3%)	
< 65 years	17 (100%)	335 (82.1%)	81 (40.7%)	504 (31.1%)	
≥ 65 years	0 (0%)	73 (17.9%)	118 (59.3%)	1115 (68.9%)	
Target organ damage					
Proteinuria	0 (0%)	0 (0%)	0 (0%)	273 (16.9%)	< 0.001
eGFR ≤ 30 ml/min/1.73 m^2^	0 (0%)	0 (0%)	0 (0%)	185 (11.4%)	< 0.001
Retinopathy	0 (0%)	0 (0%)	0 (0%)	263 (16.2%)	< 0.001
Any target organ damage	0 (0%)	0 (0%)	0 (0%)	582 (35.9%)	ND
Target organ damage, N. of target organs involved					< 0.001
0	17 (100%)	408 (100%)	199 (100%)	1037 (64.1%)	
1	0 (0%)	0 (0%)	0 (0%)	461 (38.5%)	
2	0 (0%)	0 (0%)	0 (0%)	103 (6.4%)	
3	0 (0%)	0 (0%)	0 (0%)	18 (1.1%)	
CV risk factors					
Active smoking	0 (0%)	39 (9.6%)	6 (3.0%)	258 (15.9%)	< 0.001
Dyslipidemia	0 (0%)	165 (40.4%)	76 (38.2%)	1236 (76.3%)	< 0.001
Arterial hypertension	0 (0%)	186 (45.6%)	100 (50.3%)	1414 (87.3%)	< 0.001
BMI ≥ 30 kg/m^2^	0 (0%)	111 (27.2%)	31 (15.6%)	701 (43.3%)	< 0.001
CV risk factors, N. of risk factors					< 0.001
0	17 (100%)	43 (10.5%)	0 (0%)	27 (1.7%)	
1	0 (0%)	156 (38.2)	67 (33.7%)	122 (7.5%)	
2	0 (0%)	209 (51.2%)	132 (66.3%)	275 (17%)	
3	0 (0%)	0 (0%)	0 (0%)	778 (48.1%)	
4	0 (0%)	0 (0%)	0 (0%)	367 (22.7%)	
5	0 (0%)	0 (0%)	0 (0%)	50 (3.1%)	
Current treatment					
Metformin	12 (70.1%)	322 (79.9%)	148 (74.4%)	1109 (68.5%)	< 0.001
Basal insulin	2 (11.8%)	49 (12.0%)	53 (26.6%)	524 (32.4%)	< 0.001
Rapid insulin	0 (0%)	23 (5.6%)	25 (12.6%)	220 (13.6%)	0.019
Sulfonylurea	2 (11.8%)	27 (6.6%)	29 (14.6%)	161 (9.9%)	0.002
Pioglitazone	2 (11.8%)	13 (3.2%)	18 (9.0%)	89 (5.5%)	0.016
Repaglinide	0 (0%)	0 (0%)	0 (0%)	0 (0%)	–
Acarbose	1 (5.9%)	4 (0.1%)	3 (1.5%)	26 (1.6%)	0.385
DPP4i	3 (17.6%)	51 (12.5%)	47 (23.6%)	251 (15.5%)	0.005
GLP-1 RA	0 (0%)	41 (10.0%)	23 (11.6%)	224 (13.8%)	0.007
SGLT2i	1 (5.9%)	39 (9.6%)	26 (13.1%)	203 (12.5%)	0.312

Data are expressed as n (%). Percentages are calculated per risk category, unless otherwise specified. The Moderate-to-High category includes patients that did not meet the criteria to be included in any of the risk categories defined by the 2019 ESC/EASD guidelines. ^a^Percentage of the entire population (N = 2,243). Target organ damage: proteinuria, renal impairment defined as eGFR < 30 mL/min/1.73 m^2^, left ventricular hypertrophy, or retinopathy. Risk factors: Age, hypertension, dyslipidemia, smoking, obesity. CVD: cardiovascular disease; LV: left ventricle; DPP4i: Dipeptidyl peptidase-4 inhibitor; GLP-1 RA: glucagon-like peptide-1 receptor agonist; SGLT2i: Sodium/glucose cotransporter-2 inhibitor

Interestingly, 408 of the patients (18.2%) did not fit into any of the 3 ESC/EASD risk categories. Most of the patients in this subgroup (n = 284 [69.6%]) were > 50 years old and with T2DM duration < 10 years, while only 8.8% of them did not present any CV risk factor. Since their characteristics were intermediate between M_CVR_ and H_CVR_ categories, we classified these patients into a newly coined additional CV risk category which we called “moderate-to-high” (MH_CVR_).

### CV risk factors

41.4% (n = 671) of the patients with VH_CVR_ had a history of established CV disease, 35.9% (n = 582) other target organ damage, 16.9% (n = 273) proteinuria, 16.2% (n = 263) retinopathy, and 11.4% (n = 185) reduced eGFR (eGFR ≤ 30 ml/min/1.73 m^2^). Compared with other risk groups, patients with VH_CVR_ were more frequently smokers (15.9% vs. 3.0%, 0% and 9.6% in patients with H_CVR_, M_CVR_, and MH_CVR_, respectively; p < 0.001), dyslipidemic (76.3% vs. 38.2%, 0%, and 40.4%; p < 0.001), hypertensive (87.3% vs. 50.3, 0%, and 45.6%; p < 0.001), and obese (43.3% vs. 15.6%, 0%, and 27.2%; p < 0.001).

Younger patients (< 50 years old) were almost evenly distributed between the MH_CVR_ and VH_CVR_ categories (45.3% [n = 112] and 43.7% [n = 108] respectively), while the vast majority of those aged ≥ 65 had VH_CVR_ (85.4% [n = 1115]).

Compared with other risk groups, patients with VH_CVR_ were older, with the highest rate of age ≥ 65 (68.9% vs. 59.3%, 0%, and 17.9% in patients with H_CVR_, M_CVR_ and MH_CVR_, respectively; p < 0.001), and duration of T2DM ≥ 10 years (56.8% vs. 0%, 0%, and 4.4%; p < 0.001).

### T2DM pharmacological treatments

This analysis showed significant differences in pharmacological treatment between different CV risk groups (Table 2).

Compared with other risk groups, patients with VH_CVR_ showed the highest rate of treatment with basal insulin (32.4% vs. 26.6%, 11.8%, and 12% in patients with H_CVR_, M_CVR_, and MH_CVR_, respectively; p < 0.001), rapid insulin analogues (13.6% vs. 12.6%, 0%, and 5.6%; p = 0.019) and GLP-1 RA (13.8% vs. 11.6% vs. 0%, and 10%; p = 0.007). Notably, GLP-1 RA or SGLT-2i were prescribed only to 26.3% of the patients with VH_CVR_ and to 24.7% of those with H_CVR_.

Patients with H_CVR_ had the highest rate of treatment with DPP4i (23.6% vs. 15.5%, 17.6%, and 12.5% in patients with VH_CVR_, M_CVR_, and MH_CVR_, respectively; p = 0.005) and sulfonylureas (14.6% vs. 9.9% vs. 11.8% vs. 6.6%; p = 0.002), while patients with M_CVR_ showed the highest rate of pioglitazone utilization (11.8% vs. 5.5%, 9,0, and 3.2% in patients with VH_CVR_, H_CVR_, and MH_CVR_, respectively; p = 0.016).

### Glycaemic control

The mean HbA1c level of the study population was 7.5 ± 3.4% (58.7 ± 13.4 mmol/mol) and this analysis showed significant differences in glycaemic control in the 4 CV risk groups (p = 0.007). HbA1c level was 8.1 ± 4.1% (65.3 ± 21.6 mmol/mol) in patients with M_CVR_, 7.4 ± 3.5% (57.0 ± 14.3 mmol/mol) in patients with MH_CVR_, 7.5 ± 3.4% (58.2 ± 13.5 mmol/mol) in patients with H_CVR_, and 7.6 ± 3.3% (59.1 ± 13.1 mmol/mol) in patients with VH_CVR_ (p = 0.007).

**Fig. 1:**
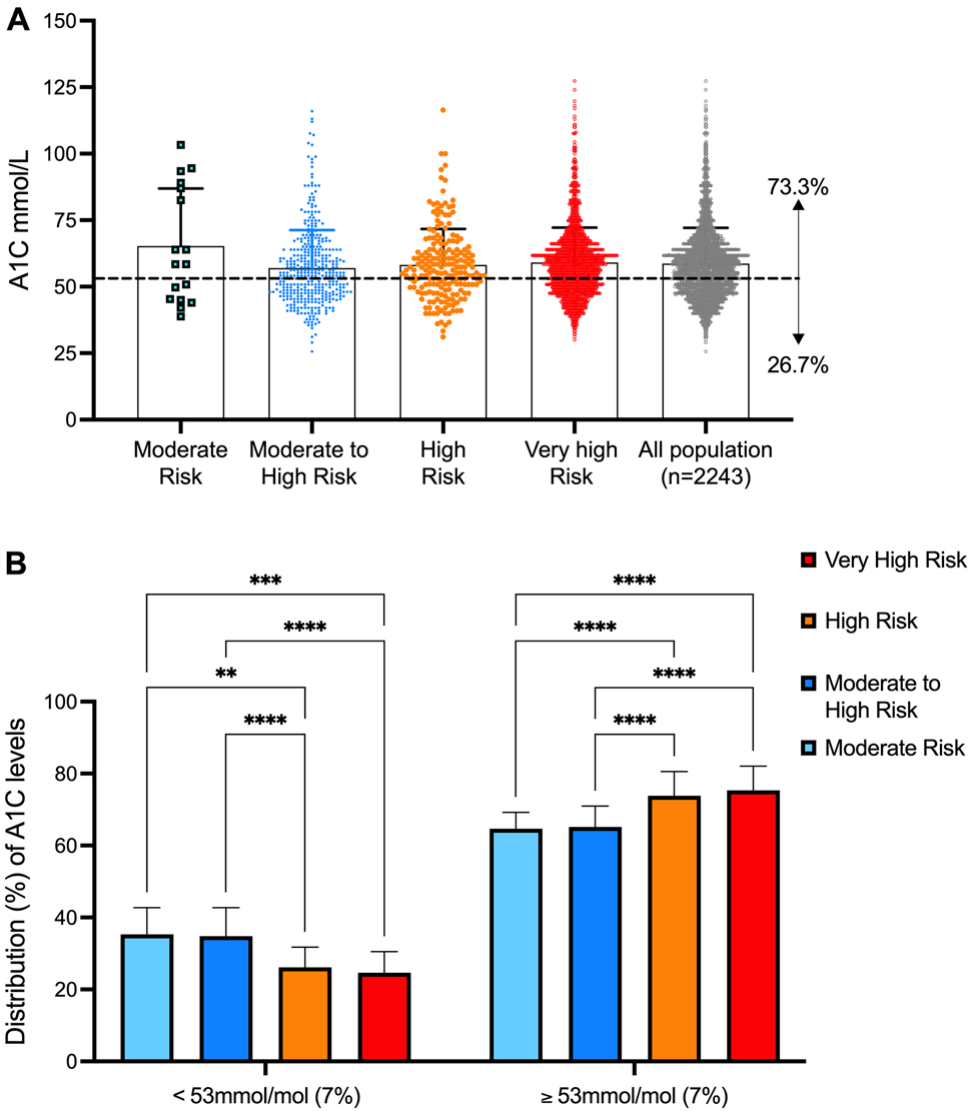
HbA1c levels distribution in the study population. **a** HbA1c values plotted as individual dots, and mean values ± 95% CI are shown for each CV risk group (moderate-to-high [light blue], moderate [blue], high [orange], very high [red]) and for the total populations (gray). The dashed line displays the 2022 ADA guidelines HbA1c target (53 mmol/mol [7%]), which was reached only by the 26.7% of the tested patients. **b** Bars represent the number (%) ± SD of patients with HbA1c levels lower (left panel) or higher (right panel) than 53 mmol/mol (7%) in moderate-to-high (light blue), moderate (blue), high (orange), and very high (red) groups, showing that patients classified in the moderate-to-high and moderate groups have statistically significant lower HbA1c levels (** = *p* < 0.01; *** = *p* < 0.001;**** = *p* < 0.0001. HbA1c: glycated hemoglobin; CI: confidence interval; CV: cardiovascular; ADA: American Diabetes Association; SD: standard deviation)

Figure 1a shows the distribution of HbA1c measurements in each CV risk group. According to the 2022 American Diabetes Association (ADA) Standards of Medical Care in Diabetes, the appropriate HbA1c target for T2DM treatment in non-pregnant adults without significant hypoglycaemia is < 7% (< 53 mmol/mol) [31]. Therefore, only 26.7% (n = 599) of the all population achieved ADA glycaemic target. In particular, the percentage of patients at target was higher (p < 0.01) in patients with M_CVD_ (35.3%) and MH_CVD_ (34.8%) as compared with H_CVD_ (26.1%) and VH_CVD_ (24.6%) (Fig. 1b).

We also evaluated whether the presence or absence of all the recorded patients’ characteristics was associated with higher or lower levels of HbA1c (Table 3). HbA1c levels were significantly higher in patients with established CVD (p = 0.041), longer diabetes duration (≥ 10 vs < 10 years; p < 0.001), younger age (< 65 vs. ≥ 65 years; p = 0.013), retinopathy (p < 0.001), dyslipidemia (p < 0.001), arterial hypertension (p = 0.007), and obesity (BMI ≥ 30 kg/m^2^; p < 0.001).

**Table 3:** Mean HbA1c levels ± SD by patients’ characteristics

Patients’ characteristics	Absent		Present		P-value
	%	mmol/mol	%	mmol/mol	
History of established CVD	7.5 ± 3.4	58.3 ± 13.6	7.6 ± 3.3	59.6 ± 13.0	0.041
Diabetes duration ≥ 10 years	7.4 ± 3.4	57.3 ± 13.8	7.6 ± 3.3	60.1 ± 12.9	< 0.001
Age					
Age ≥ 50 years	7.6 ± 3.5	59.3 ± 14.7	7.5 ± 3.4	58.6 ± 13.5	0.492
Age ≥ 65 years	7.6 ± 3.5	59.5 ± 14.8	7.5 ± 3.3	58.1 ± 12.4	0.013
Target organ damage					
Proteinuria	7.5 ± 3.4	58.6 ± 13.4	7.6 ± 3.4	59.8 ± 13.6	0.148
eGFR ≤ 30 ml/min/1.73 m2	7.5 ± 3.4	58.6 ± 13.5	7.7 ± 3.3	60.4 ± 12.8	0.076
Retinopathy	7.5 ± 3.4	58.3 ± 13.6	7.8 ± 3.3	61.4 ± 12.1	< 0.001
Target organ damage (N. of organs involved)	7.5 ± 3.4	58.2 ± 13.7	7.7 ± 3.3	60.2 ± 12.7	0.003
0	7.5 ± 3.4	58.2 ± 13.7			0.003
1			7.6 ± 3.3	59.8 ± 12.4	
2			7.7 ± 3.4	60.7 ± 13.6	
3			8.2 ± 3.4	66.6 ± 13.5	
CV risk factors					
Active smoking	7.5 ± 3.4	58.6 ± 13.4	7.6 ± 3.4	59.3 ± 13.4	0.408
Dyslipidemia	7.4 ± 3.4	57.0 ± 13.5	7.6 ± 3.4	59.6 ± 13.4	< 0.001
Arterial hypertension	7.4 ± 3.4	57.4 ± 13.4	7.6 ± 3.4	59.1 ± 13.4	0.007
Obesity	7.4 ± 3.3	57.6 ± 12.8	7.7 ± 3.5	60.6 ± 14.3	< 0.001
N. of CV risk factors					
0	7.3 ± 3.6	56.7 ± 16.1			0.001
1			7.4 ± 3.3	57.1 ± 12.3	
2			7.5 ± 3.4	58.3 ± 13.7	
3			7.5 ± 3.4	58.7 ± 13.7	
4			7.7 ± 3.3	61.0 ± 12.7	
5			7.8 ± 3.2	61.6 ± 11.7	
≥ 3	7.3 ± 3.6	56.7 ± 16.1	7.6 ± 3.4	59.5 ± 13.4	0.061
Current treatment					
Metformin	7.5 ± 3.4	58.5 ± 13.6	7.5 ± 3.4	58.8 ± 13.4	0.617
Basal insulin	7.3 3.3	56.5 ± 12.8	8.0 ± 3.4	64.3 ± 13.4	< 0.001
Rapid insulin	7.4 ± 3.3	57.7 ± 12.9	8.2 ± 3.6	65.8 ± 15.3	< 0.001
Sulfonylurea	7.5 ± 3.4	58.2 ± 13.3	7.9 ± 3.4	63.2 ± 13.5	< 0.001
Pioglitazione	7.5 ± 3.4	58.6 ± 13.5	7.6 ± 3.2	60.0 ± 11.8	0.277
Repaglinide	7.5 ± 3.4	58.7 ± 13.4	0.0 ± 0.0	00.0 ± 0.00	ND
Acarbose	7.5 ± 3.4	58.7 ± 13.5	7.6 ± 3.0	59.5 ± 9.5	0.737
DPP4i	7.6 ± 3.4	59.1 ± 13.8	7.3 ± 3.2	56.6 ± 11.2	0.002
GLP-1 RA	7.5 ± 3.4	59.0 ± 13.5	7.3 ± 3.4	56.8 ± 13.0	0.010
SGLT2i	7.5 ± 3.4	59.0 ± 13.7	7.3 ± 3.2	56.7 ± 11.4	0.008

N = 2243 patients. ND = data not available. Target organ damage: proteinuria, renal impairment defined as eGFR ≤ 30 mL/min/1.73 m^2^, left ventricular hypertrophy, or retinopathy. Risk factors: Age, hypertension, dyslipidemia, smoking, obesity. HbA1c: glycated haemoglobin; CVD: cardiovascular disease; LV: left ventricle; DPP4i: Dipeptidyl peptidase-4 inhibitor; GLP-1 RA: glucagon-like peptide-1 receptor agonist; SGLT2i: Sodium/glucose cotransporter-2 inhibitor

HbA1c levels were significantly lower in patients without CV risk factors compared with subjects with at least one risk factor (p = 0.001), with glycaemic control progressively worsening with the increase of risk factors number.

Similarly, the presence of target organ damage was associated with higher HbA1c levels (p = 0.003), with worsening of glycaemic control being associated with the increased number of affected organs.

Finally, the type of pharmacological treatment seemed to be associated with different glycaemic control. Patients treated with insulin or sulfonylureas showed higher HbA1c values (p < 0.001) as compared with patients treated with the newer antidiabetic medications (DPP4i, GLP-1 RA, and SGLT2i) which had better glycaemic control (p = 0.002, p = 0.010, and p = 0.008 respectively).

## Discussion

To the best of our knowledge, this is the first study to test a Web App for T2DM patients’ CV risk stratification. In our experience, the AWARE App proved to be a suitable tool for real-world clinical practice and it allowed us to assess rapidly and efficiently in more than 2,000 consecutive subjects.

The T2DM CV risk redefinition introduced in 2019 ESC/EASD guidelines, based on risk factors, organ damage, and duration of disease, places a large proportion of patients in the high and very high risk categories. This was confirmed by our study since the majority of the enrolled patients (72.2%) belonged to the VH_CVR_ group. These patients were generally ≥ 65 years old (68.9%), with a long disease duration (≥ 10 years [56.8%]), a history of established CV disease (41.4%) and organ damage (35.5%). As expected, patients with VH_CVR_ also showed higher rates of hypertension, dyslipidaemia and obesity as compared with other risk groups.

Interestingly, about 18% of the patients in this study did not fit in any of the three 2019 ESC/EASD CV risk categories and we included them in the newly coined MH_CVR_ group. The majority of the subjects in this group had shorter disease duration (< 10 years) and younger age (50–65 years). None of them had a history of CV disease or was affected by target organ damage such as retinopathy, proteinuria and advanced kidney disease. However, almost 90% of the MH_CVR_ patients had one or more CV risk factors, and their rates of hypertension, dyslipidaemia, and obesity were higher compared with those of patients with H_CVR_. In our opinion, this relatively young population, with short disease duration, less than 3 CV risk factors, that has not already developed any retinal or cardio-renal complication, may also greatly benefit from newer therapies such as GLP-1 RA and SGLT2i, which have demonstrated to reduce CV diseases risk and mortality [32, 33].

This study results are consistent with published data. A recent prospective study conducted on 1,690 T2DM patients compared the prognostic performance of the 2019 ESC/EASD CV risk model with the Systematic COronary Risk Evaluation (SCORE) risk model and N-terminal pro-B-type natriuretic peptide (NT-proBNP) levels measurement [34]. The high rate of patients belonging to the 2019 ESC/EASD very high risk category and the rate of subjects who could not be categorized according to these criteria were similar to our findings (66% and 17%, respectively). Interestingly, the uncategorized patients’ characteristics were similar to those of our MH_CVR_ subjects in terms of clinical features, such as disease duration (< 10 years) and number of CV risk factors (< 3).

In an Italian, retrospective study, which evaluated 473,740 T2DM patients, the rate of very high CV risk subjects based on 2019 ESC/EASD criteria resulted similar to our findings (78.5%) [35]. The characteristics of this subgroup of patients were consistent with our data (long disease duration, history of CV events, high rates of end-organ damage and ≥ 3 CV risk factors). According to the authors of this study, T2DM is not always an equivalent of CV risk (i.e. T2DM patients without coronary artery disease [CHD] and patients with only CHD have similar mortality rates); in fact, it could exist a subgroup of younger patients with shorter disease duration and with low risk of CV events [36]. This subgroup of T2DM patients, with features similar to those of our MH_CVR_ subjects, could not be categorized employing the 2019 ESC/EASD criteria, and may therefore not receive the most appropriate treatment.

Even though knowing the CV risk level is essential for choosing the most appropriate treatment in T2DM patients, the degree of glycometabolic control is critically important. This is the reason why we decided to include in the AWARE App form also the HbA1c level (which is not included in the 2019 ESC/EASD criteria). In our study population, glycaemic control was unsatisfactory, with HbA1c level of 7.5 ± 3.4% (58.7 ± 13.4 mmol/mol) and a high proportion (almost 3/4) of patients with HbA1c values above the 2022 ADA Standard of medical Care in Diabetes and 2019 ESC/EASD guidelines recommended target (< 53 mmol/mol, < 7%) [26, 31]. This result may be in part due to delayed choice of treatment (as shown by the low rate of patients with VH_CVR_ treated with GLP-1 RA or SGLT2i), and it is consistent with the findings of the CAPTURE study. In this multinational, cross-sectional trial, conducted in 9,823 T2DM patients and 13 different countries, the median HbA1c level was 7.3% (6.6–8.4%) (56 mmol/mol [49–68 mmol/mol]) and only 21.5% of the patients with established CV disease were treated with GLP-1 RA of SGLT2i [37].

The unsatisfactory glycaemic control reported in our study may also reflect a cultural legacy stemming from trials such as ACCORD and VADT, which demonstrated that intensive glycaemic control in T2DM patients does not provide any significant benefit and can even increase mortality [38]. It should be highlighted, though, that ACCORD and VADT patients were older, with longstanding diabetes, and a great prevalence of macrovascular disease; in subjects with these characteristics (which are similar to those of the VH_CVR_ group in our study), strictly pursuing near normal glucose levels with insulin could increase the frequency of hypoglycaemic events, a strong risk factor for CV acute complications and sudden death. On the contrary, UKPDS clearly showed that younger patients, with early diabetes and no overt CD disease (similar to those of our MH_CVR_ group), can greatly benefit from more aggressive glucose management in terms of long-term reduction of myocardial infarction, death from any cause, and microvascular disease[38]. Moreover, ACCORD, VADT and UKPDS results were obtained with conventional antidiabetic drugs and the discovery of newer treatments requires a re-appraisal of those findings. Thanks to the intrinsic low risk of hypoglycaemia associated with GLP-1 RA of SGLT2i, these drugs allow to safely achieve the recommended HbA1c levels, even in patients with advanced diabetes, thus allowing more aggressive treatment of T2DM. Recently, several small studies in newly diagnosed T2DM patients showed that the use of a combination of multiple drugs with complementary mechanisms of action (metformin, pioglitazone, and exenatide) provides better outcomes compared with the sequential treatment with conventional medications [39–41].

In conclusion, we believe that the use of the web app AWARE to evaluate CV risk and implement more aggressive earlier treatment with newer medications could represent a step forward to help preventing chronic severe and invalidating complications and premature death in T2DM. These hypothesis should be verified by larger, prospective trials, in order to possibly overcome two limitations of our study, i.e. 1) its retrospective design and 2) a sample of patients belonging to a restricted geographical region.

## Conclusion

The AWARE App represents a practical tool for a very rapid CV risk stratification of T2DM patients, with the potential to increase physicians’ awareness of this important patient feature, to guide them in the choice of the best therapeutic option, and improve their adherence to current treatment guidelines. In our population, the stratification with the AWARE App showed a vast majority of T2DM patients with very high CV risk and a relevant subgroup (about 20%) who did not fit in any 2019 ESC/EASD category. Moreover, it showed low treatment’s rates with newer T2DM drugs (GLP-1 RA and SGLT2i) in patients with high or very high CV risk which might benefit from these treatments.

## Supplementary Information

Below is the link to the electronic supplementary material.

Supplementary file1 (PDF 616 KB)

## Data Availability

The dataset analysed in the current study will be made available by the corresponding authors upon reasonable request.
