# Supplementary material for: AWARE A novel web application to rapidly assess cardiovascular risk in type 2 diabetes mellitus
Source: Acta Diabetol. 2023 Jun 4;60(9):1257–66. doi: 10.1007/s00592-023-02115-x (PMC10359387; doi:10.1007/s00592-023-02115-x)
Supplement: Supplementary file 1 — Supplementary file1 (PDF 616 KB) [file 592_2023_2115_MOESM1_ESM.pdf]

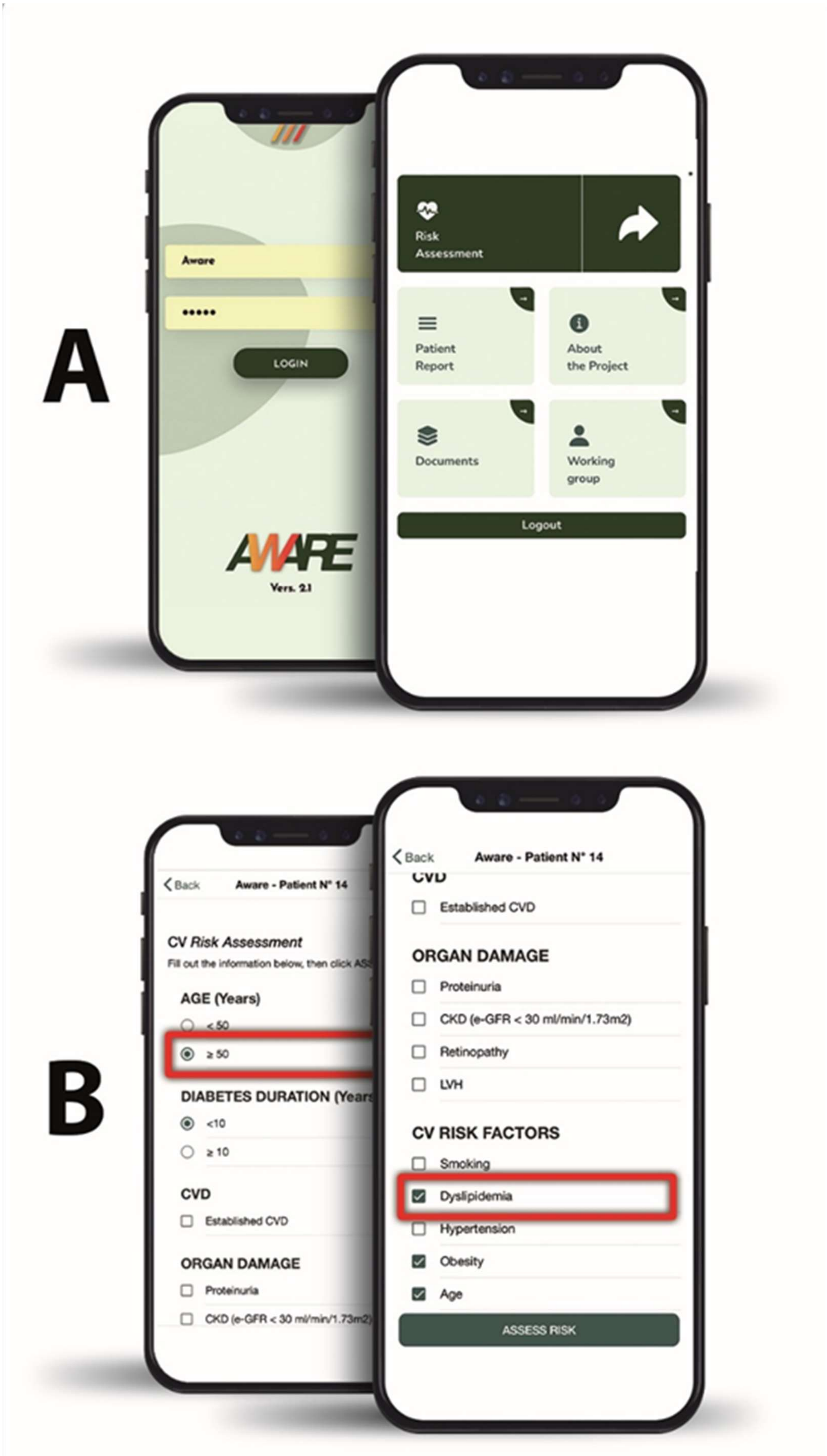

A
Aware
LOGIN
AWARE
Vers. 2.1
Risk Assessment
Patient Report
About the Project
Documents
Working group
Logout
B
Back
Aware - Patient N° 14
CV Risk Assessment
Fill out the information below, then click ASS
AGE (Years)
< 50
≥ 50
DIABETES DURATION (Years
<10
≥ 10
CVD
Established CVD
ORGAN DAMAGE
Proteinuria
CKD (e-GFR < 30 ml/min/1.73m2)
Retinopathy
LVH
CV RISK FACTORS
Smoking
Dyslipidemia
Hypertension
Obesity
Age
ASSESS RISK

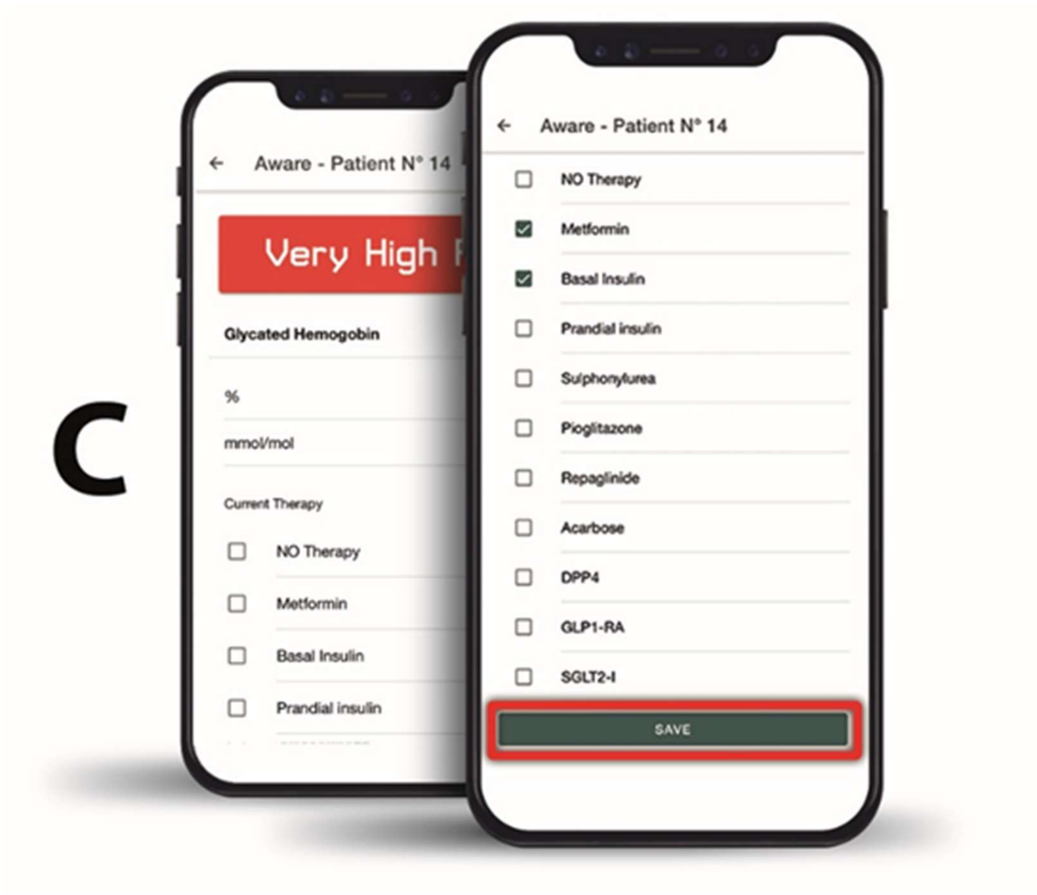

Supplementary Fig. 1 The AWARE App user interface. The App is free and available online without registration at the following URL: https://aware.softwarevm.online/ (user ID: Aware; password: Aware). A) Login screen (left) and Main menu (right), which allows to access the CV risk assessment section and other informative sections. B) Form for CV risk assessment, based on 2019 ESC/EASD criteria. C) Result screen, which returns the CV risk category and allows to record additional patient data (HbA1c level and type of treatment).
